# Supplementary material for: Electrochemically induced hyperfluorescence based on the formation of charge-transfer excimers
Source: Nat Commun. 2026 Mar 10;17:3753. doi: 10.1038/s41467-026-70291-9 (PMC13106640; doi:10.1038/s41467-026-70291-9)
Supplement: Supplementary file 1 — Supplementary Information [file 41467_2026_70291_MOESM1_ESM.pdf]

Supplementary Information

Electrochemically induced hyperfluorescence based on the formation of charge-transfer excimers

Chang-Ki Moon[1,2], Yuka Yasuda[3], Yu Kusakabe[3], Anna Popczyk[1], Shohei Fukushima[3], Julian Butscher[1], Nachiket Pathak[1], Joel Schlecht[4], Kuraudo Ishihara[3], Oliver Dumele[4], Hironori Kaji[3]*, Malte C. Gather[1,2]*

[1]Humboldt Centre for Nano- and Biophotonics, Institute for Light and Matter, Department of Chemistry and Biochemistry, University of Cologne, Greinstr. 4-6, 50939 Köln, Germany

[2]Organic Semiconductor Centre, School of Physics and Astronomy, University of St Andrews, North Haugh, St Andrews KY16 9SS, United Kingdom

[3]Institute for Chemical Research, Kyoto University, Uji, Kyoto 611-0011, Japan

[4]Institute of Organic Chemistry, Department of Chemistry and Biochemistry, University of Cologne, Greinstr. 4-6, 50939 Köln, Germany

*Corresponding authors: kaji@scl.kyoto-u.ac.jp; malte.gather@uni-koeln.de

This file includes:

Supplementary Figures 1-17

Supplementary Tables 1-3

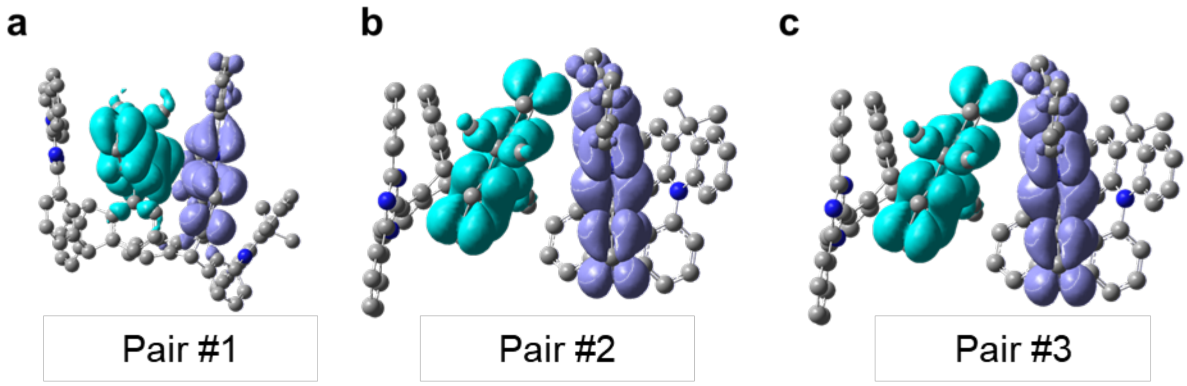

Supplementary Fig. 1 Differential charge density distributions ($\Delta\rho$) of the three cation–anion pairs of TpAT-tFFO for the $S_0 \rightarrow S_1$ transition. In all molecular pairs, the left side represents the cation and the right side represents the anion. Blue and purple indicate negative and positive $\Delta\rho$, respectively. The monomer structures were optimized using PBE0/6-31G(d) level of theory using the polarizable continuum model in toluene. A dielectric constant of 18.40 was used for the mixture of toluene and acetonitrile. MD simulations were performed on 50 systems to reproduce high concentration conditions (80 mM of TpAT-tFFO in toluene/acetonitrile (2:1 vol%). The molecular ratio of TpAT-tFFO:toluene:acetonitrile was set to 8:627:639 molecules with half of the TpAT-tFFO molecules assigned as cations and the other half as anions. The MD simulations were performed in the NVT ensemble for 1.0 ns at 300 K with a box length of 5.5 nm. The S_1 and T_1 energies and the differential charge density distributions ($\Delta\rho$'s) were calculated for three cation-anion aggregated pairs of TpAT-tFFO using the TD-PBE0/6-31G(d) method using the polarizable continuum model in toluene and a dielectric constant of 18.40. Molecular structure optimizations and TD-DFT calculations were performed by the Gaussian 16 program package[2]. The initial arrangements of MD simulations were performed by the Packmol package[3]. The MD simulations were performed by the LAMMPS GPU package[4]. The calculations of $\Delta\rho$'s were performed by the Multiwfn package[5].

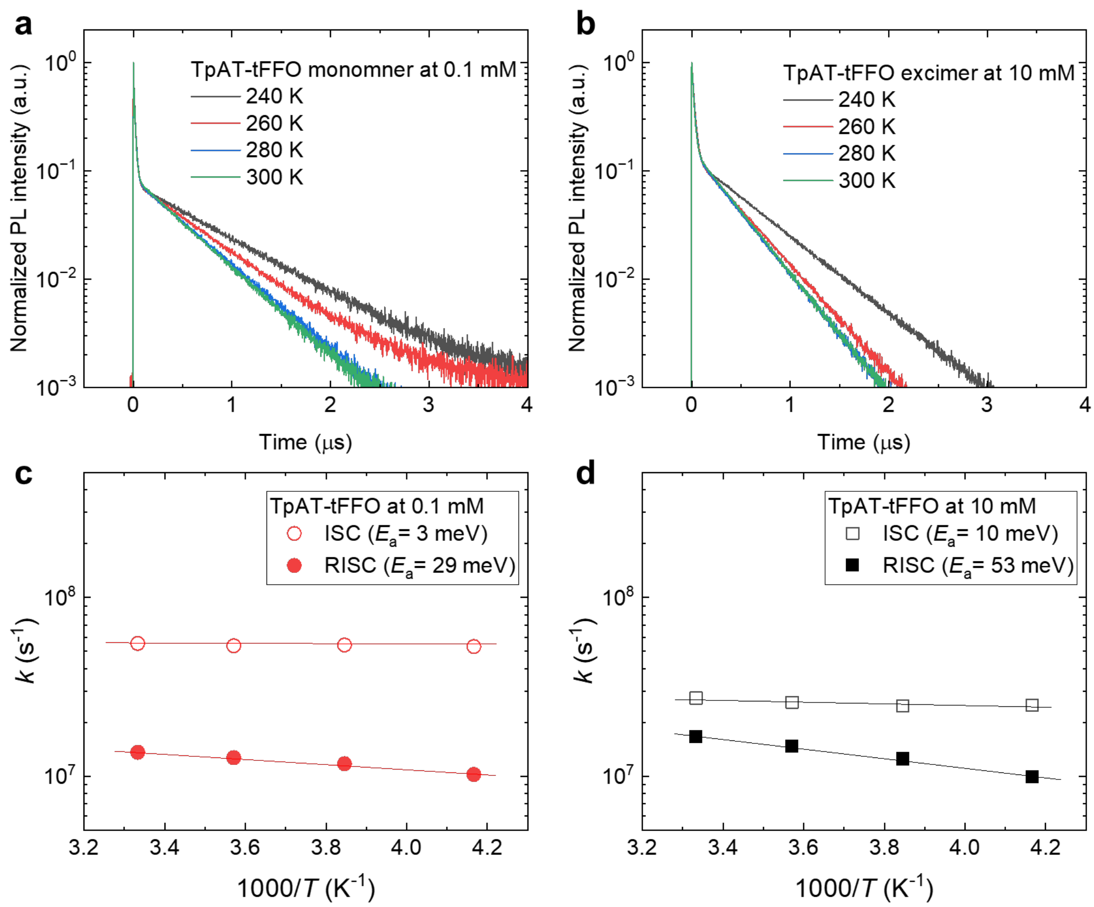

Supplementary Fig. 2. TADF analysis of TpAT-tFFO solution. Variable-temperature photoluminescence for **a** monomer emission from a solution at 0.1 mM and **b** excimer emission from a solution at 10 mM. **c,d** Analysis of k_{ISC}, k_{RISC}, and activation energy (E_a) for monomer and excimer emission.

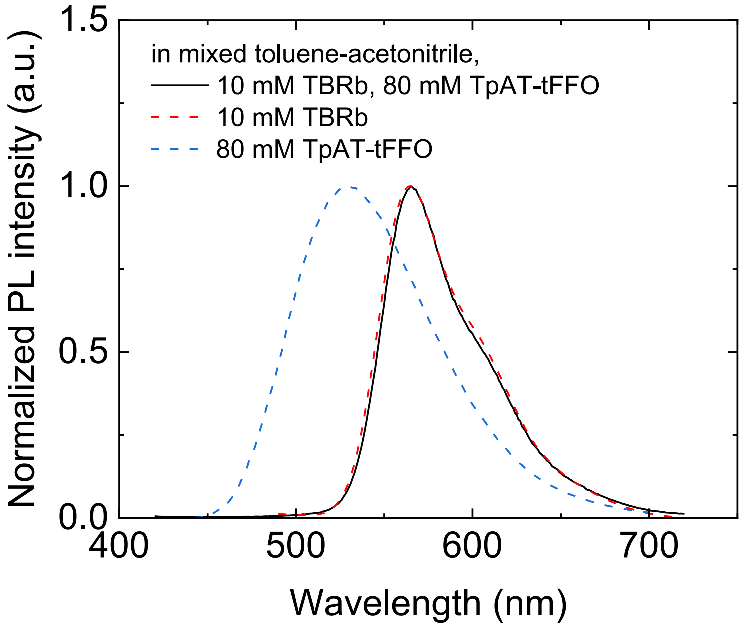

Supplementary Fig. 3. PL spectra of the mixed toluene-acetonitrile solution containing 10 mM of TBRb and 80 mM of TpAT-tFFO, both together and individually. Both TBRb and TpAT-tFFO molecules are excited by λ=373 nm light. In the mixture of TBRb and TpAT-tFFO, the emission is solely from TBRb due to rapid energy transfer from TpAT-tFFO excimers to TBRb molecules.

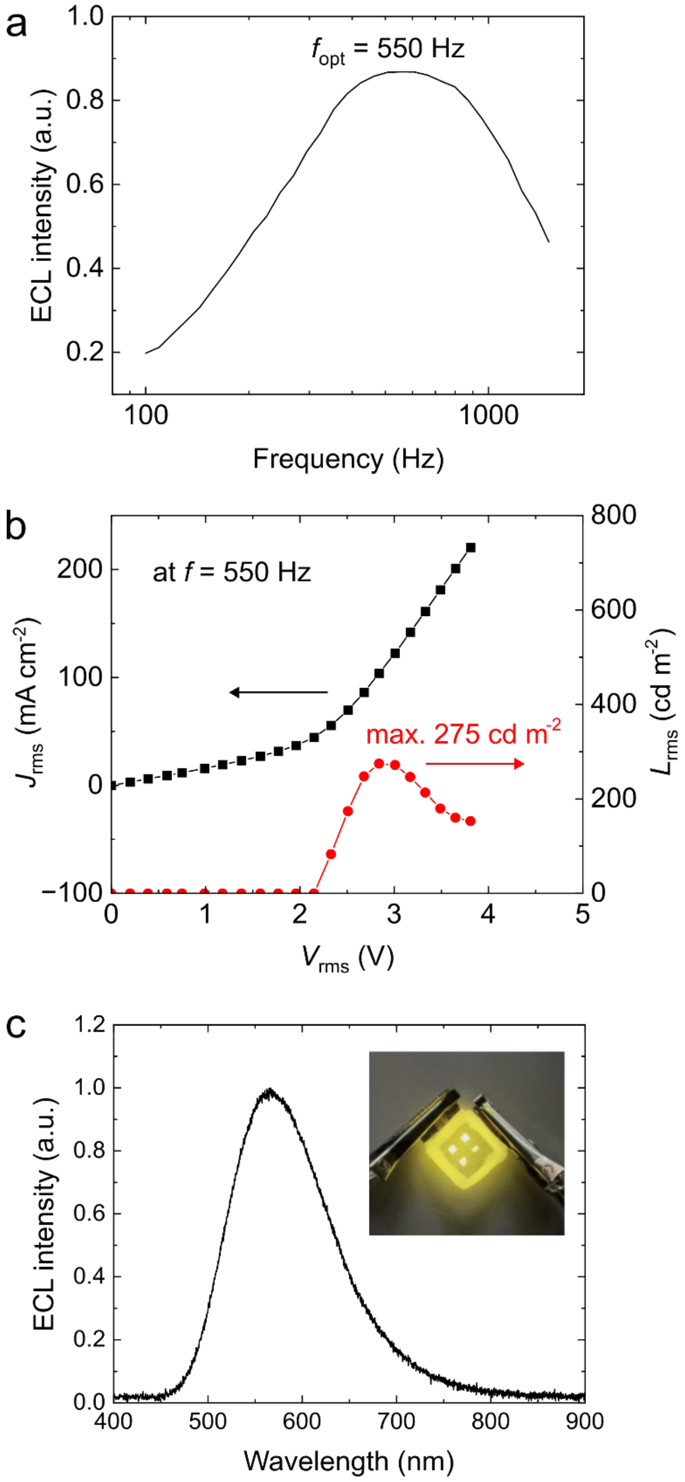

Supplementary Fig. 4. CT excimer emission ECLD. Parallel electrode (PE) structure device with two ITO-coated glass substrates and a mixed acetone and acetonitrile ECL solution containing 80 mM TpAT-tFFO and 100 mM electrolyte. **a** Frequency-dependent ECL intensity measured under AC operation. **b** Current-voltage-luminance characteristics measured at a frequency of 500 Hz. **c** ECL spectrum and photograph of the device in operation.

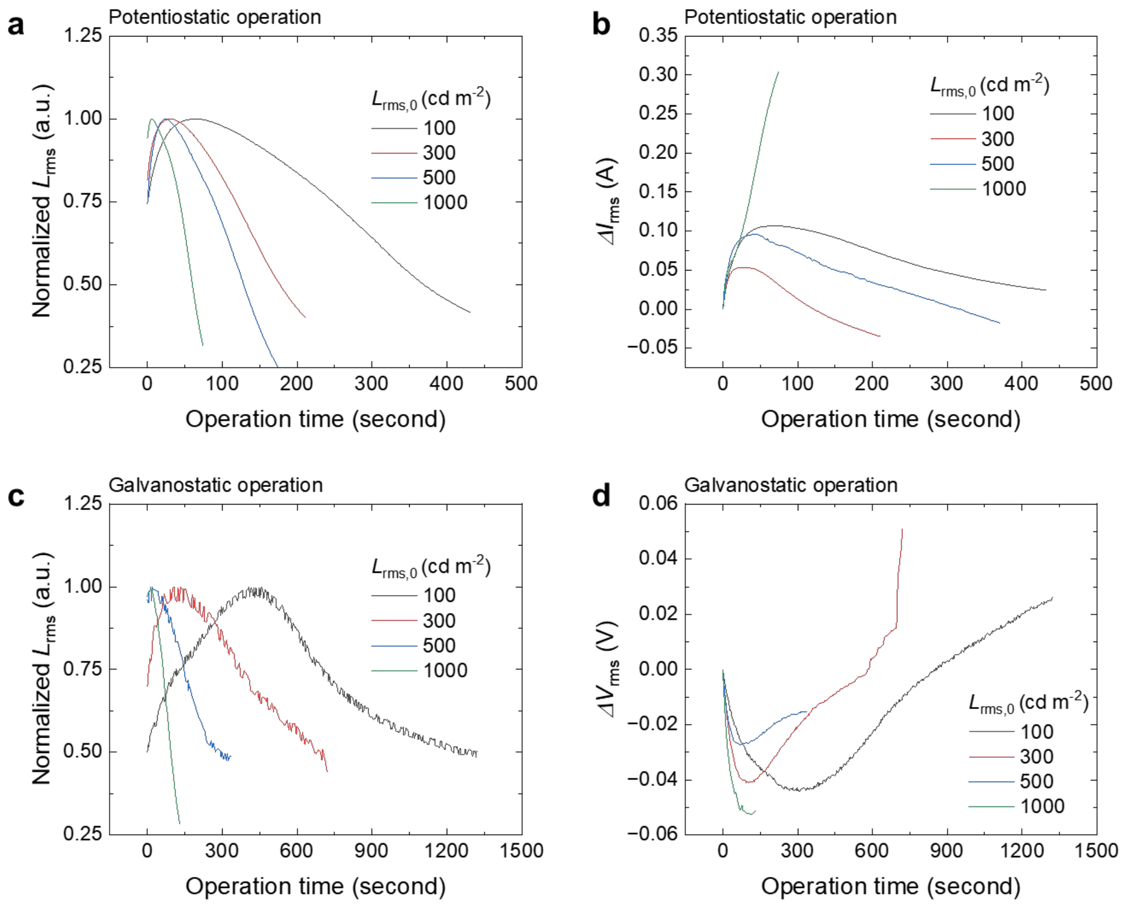

Supplementary Fig. 5. Measurement of operational lifetime of the TpAT-tFFO device. a Luminance over time under potentiostatic operation at various initial luminance values and **b** corresponding changes in the rms current. **c** Luminance over time under galvanostatic operations and **d** changes in the voltage applied to maintain the preset current. Voltage application leads to re-distribution of molecules and ions within the liquid layer. This in turn results in an initial increase in luminance along with an increase in device current under potentiostatic operation and decrease in voltage under the galvanostatic operation. The LT_{50} value was estimated as the time when the brightness decreased by half of the maximum value for each operation condition.

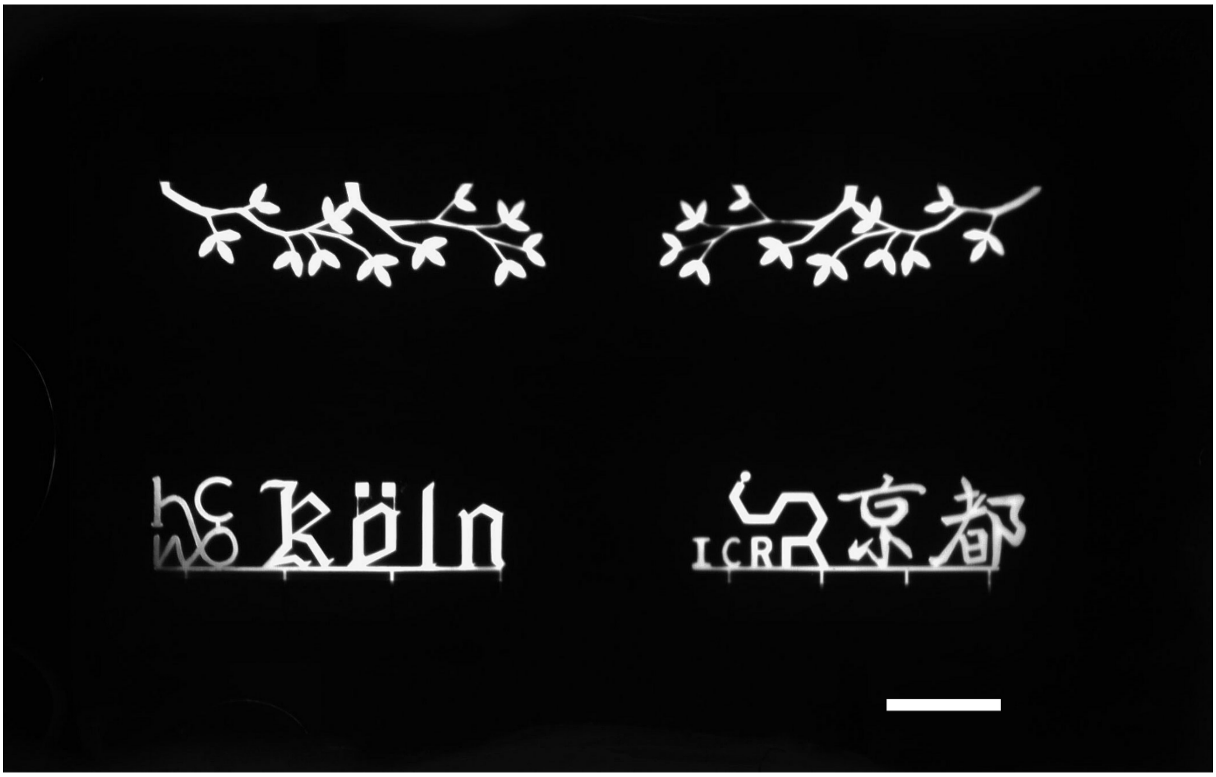

Supplementary Fig. 6. High-resolution microscope image showing the light intensity profile in greyscale from a calligraphic ECLD display configured in floating bipolar electrode (FBE) configuration. The scale bar at the bottom right represents a length of 2 mm.

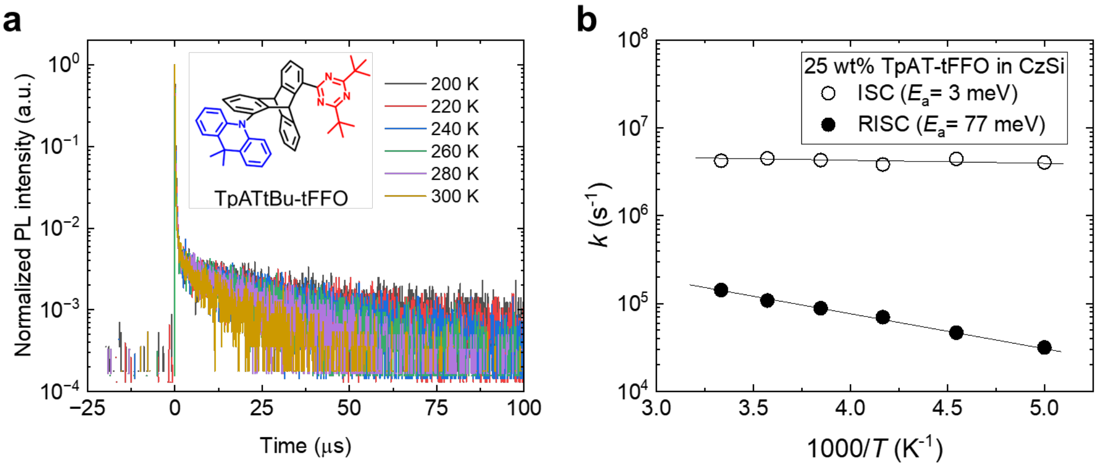

Supplementary Fig. 7. Temperature-dependent PL of a thin film of 25 wt% TpATtBu-tFFO in CzSi. a Time-resolved PL decay measured across temperatures from 200 K to 300K. **b** Temperature dependence of the rates of intersystem crossing (k_{ISC}) and reverse intersystem crossing (k_{RISC}), along with the activation energy as determined from Arrhenius fits to the data. At room temperature, the k_{ISC} and k_{RISC} rates were 4.2×10^6 s^{-1} and 1.4×10^5 s^{-1}, respectively.

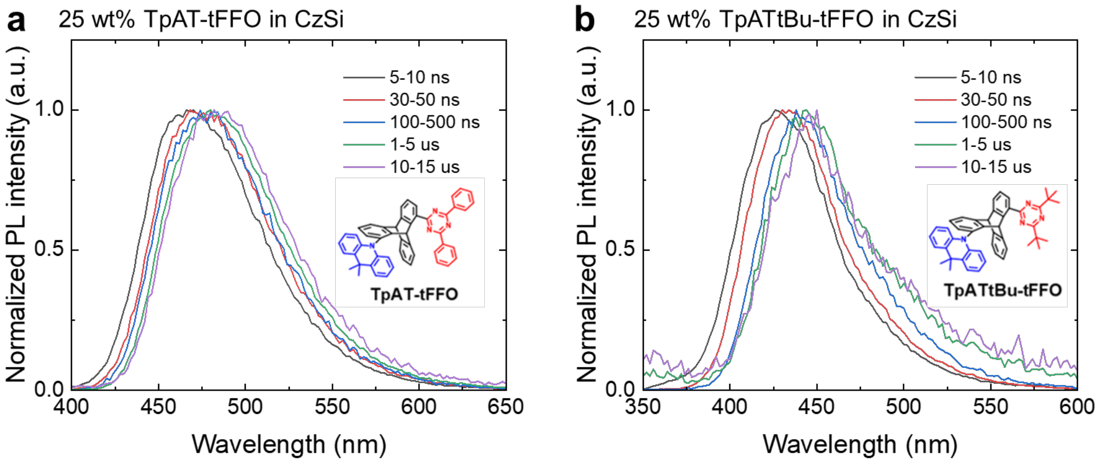

Supplementary Fig. 8. Temporal evolution of thin-film photoluminescence spectrum post excitation. Measurements for **a** TpAT-tFFO and **b** TpATtBu-tFFO doped in CzSi films show that both prompt and delayed fluorescence originate from the same singlet excited state. The observed red shift is attributed to solid-state solvation effects on the charge-transfer states.

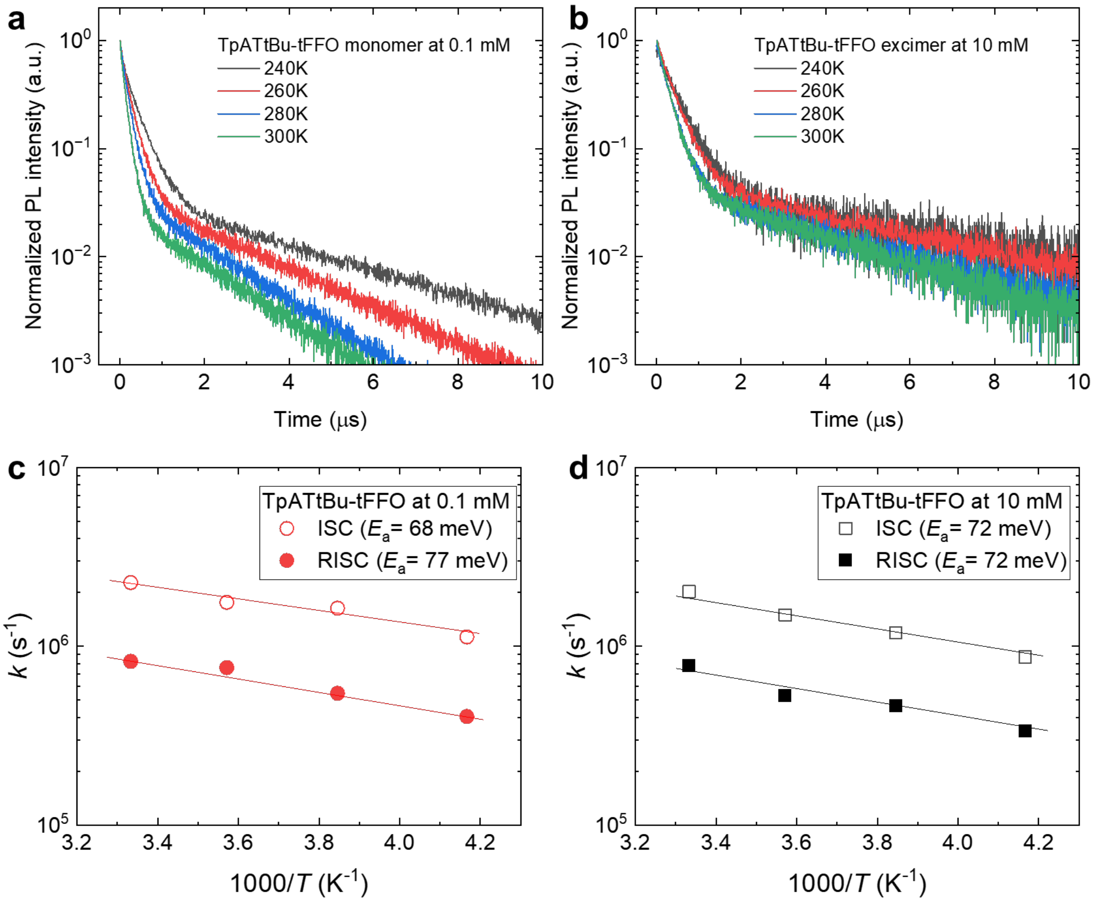

Supplementary Fig. 9. TADF analysis of TpATtBu-tFFO solution. Variable-temperature photoluminescence for **a** monomer emission from a solution at 0.1 mM and **b** excimer emission from a solution at 10 mM. **c,d** Analysis of k_{ISC}, k_{RISC}, and E_a for monomer and excimer emission.

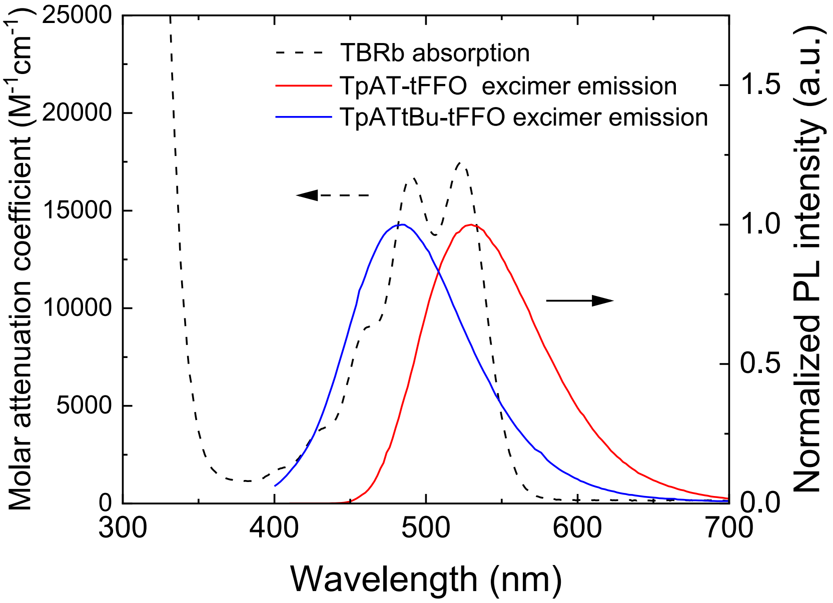

Supplementary Fig. 10. Absorption spectrum of TBRb and emission spectra of the TpAT-tFFO and TpATtBu-tFFO excimers.

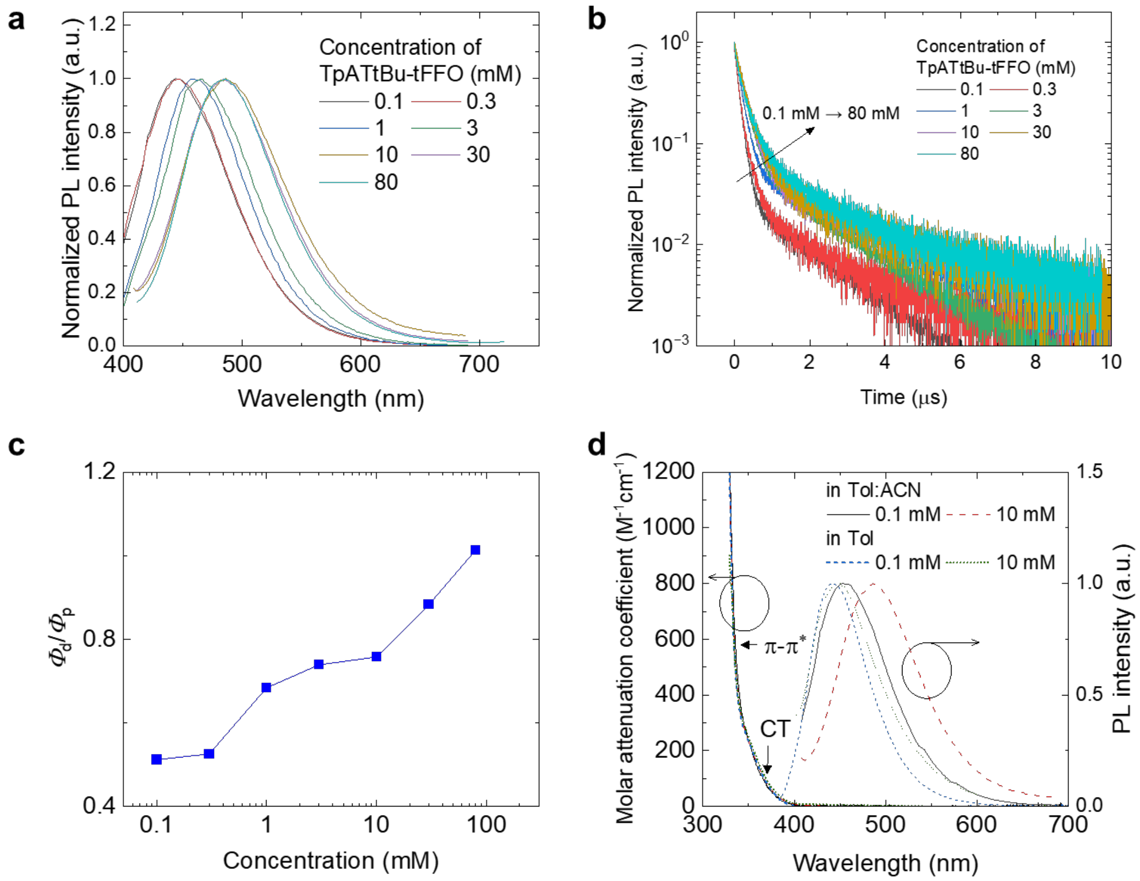

Supplementary Fig. 11. Photoluminescence characteristics of TpATtBu-tFFO solutions. a PL spectra, **b** transient PL, and **c** PLQY ratio of delayed fluorescence to prompt fluorescence of TpATtBu-tFFO for various concentrations in mixed toluene-acetonitrile solutions (2:1 by volume). **d** Molar attenuation coefficient of TpATtBu-tFFO at concentrations of 0.1 mM and 10 mM, measured in a toluene:acetonitrile mixture and pure toluene.

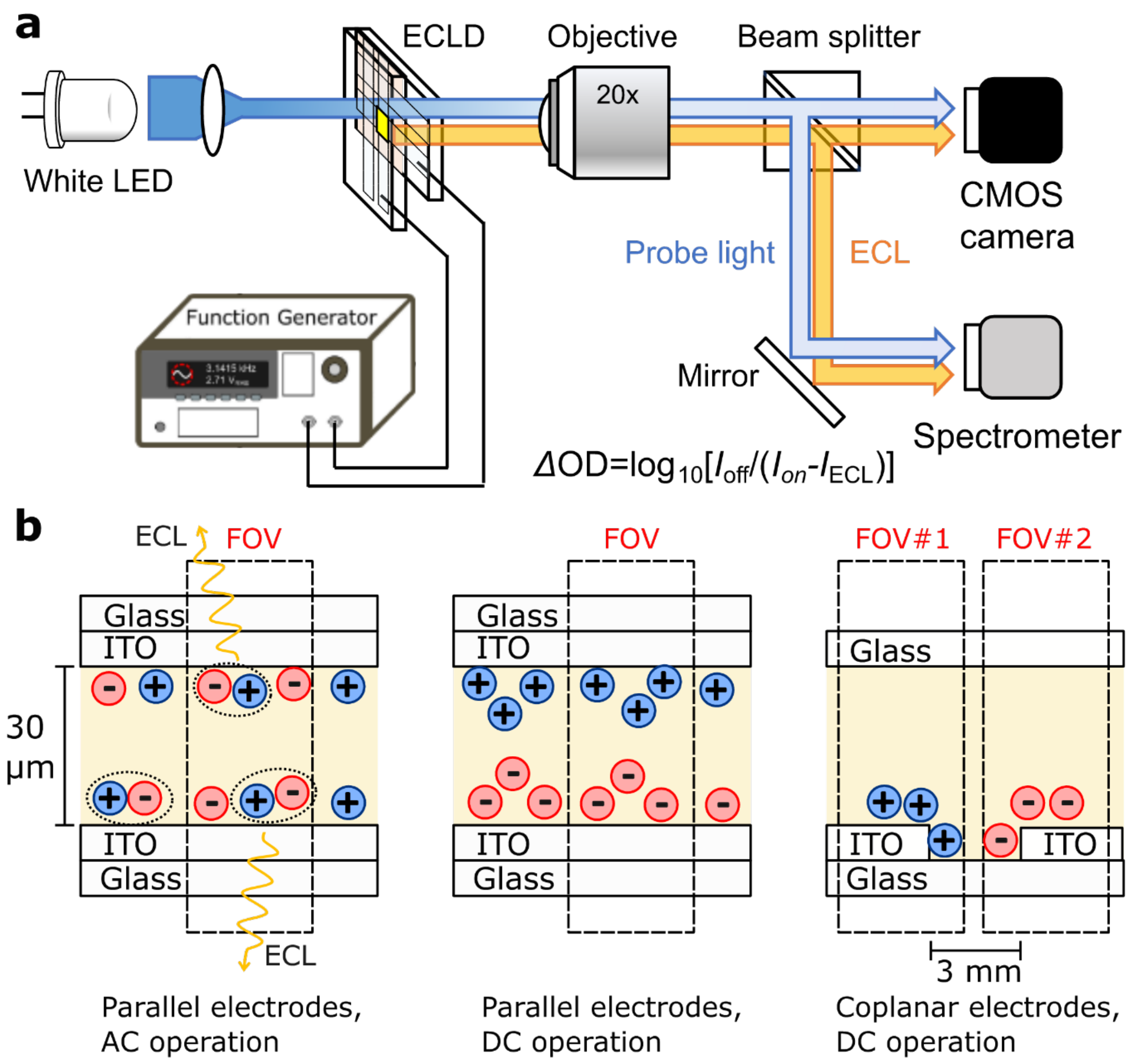

Supplementary Fig. 12. a Schematic of the absorption spectroelectrochemistry setup. **b** Device configurations, operating methods, and fields of view (FOVs) used to analyze the ion absorption bands. Under AC operation significant electroluminescence (ECL) is generated, whereas DC operation involves little ECL. For the parallel electrode configuration, cations and anions coexist within the FOV, while the coplanar electrode configuration allows to measure the absorption bands of each cation and anion separately.

Supplementary Table 1. Calculated S_1 energies, S_1(monomer)−S_1(agg. pair) energy differences, T_1 energies and ΔE_{ST} of the cation–anion aggregated pairs of TpAT-tFFO shown in Supplementary Figure 1, where agg. pair denotes an aggregated pair.

	S_1 / eV	S_1(monomer)−S_1(agg. pair) / eV	T_1 / eV	ΔE_{ST} / meV
Monomer	2.849	-	2.837	12
Agg. pair #1	2.381	0.468	2.373	8
Agg. pair #2	2.328	0.521	2.321	7
Agg. pair #3	2.490	0.359	2.487	3

Supplementary Table 2. TADF parameters of TpAT-tFFO at various concentrations in mixed toluene and acetonitrile solutions. The lifetime parameters are extracted from transient PL curves using the equation, $I(t) = I_p \exp(-t/\tau_p) + I_d \exp(-t/\tau_d)$. The analysis of k_{ISC} and k_{RISC} was performed using the method described in Methods section of reference[1].

Concentration (mM)	State	I_p	τ_p (ns)	I_d	τ_d (ns)	Φ_d/Φ_p	Φ_{PL}	k_{ISC} (10^7 s^{-1})	k_{RISC} (10^7 s^{-1})	$k_{\mathrm{ISC}}k_{\mathrm{RISC}}$ (10^{14} s^{-1})
0.1	Monomer	0.837	17.3	0.144	977	9.73	0.84	4.02	1.42	5.70
0.3	Monomer	0.883	18.3	0.128	1150	9.16	0.84	4.03	1.07	4.31
1	Mixed	0.837	20.2	0.158	928	8.65	-	3.26	1.41	4.61
3	Mixed	0.831	19.6	0.149	492	4.52	-	3.11	1.51	4.69
10	Mixed	0.838	20.4	0.158	355	3.27	-	2.66	1.69	4.49
30	Excimer	0.837	20.7	0.157	395	3.58	0.27	2.70	1.62	4.37
80	Excimer	0.794	18.6	0.131	1770	15.7	0.27	3.76	1.20	4.51

Supplementary Table 3. TADF parameters of TpATtBu-tFFO at various concentrations in mixed toluene and acetonitrile solutions. The lifetime parameters are extracted from transient PL curves using the equation, $I(t) = I_p \exp(-t/\tau_p) + I_d \exp(-t/\tau_d)$. The analysis of k_{ISC} and k_{RISC} was performed using the method described in Methods section of reference [1].

Concentration (mM)	State	I_p	τ_p (ns)	I_d	τ_d (μs)	Φ_d/Φ_p	Φ_{PL}	k_{ISC} (10^6 s^{-1})	k_{RISC} (10^5 s^{-1})	$k_{ISC}k_{RISC}$ (10^{12} s^{-1})
0.1	Monomer	0.963	142	0.037	1.92	0.51	0.52	2.27	8.24	1.88
0.3	Monomer	0.958	161	0.042	1.95	0.53	0.52	2.03	8.26	1.68
1	Mixed	0.938	190	0.061	1.98	0.68	-	1.97	9.24	1.82
3	Mixed	0.932	212	0.068	2.17	0.74	-	1.83	8.78	1.61
10	Excimer	0.942	198	0.058	2.43	0.76	0.50	2.02	7.78	1.57
30	Excimer	0.935	239	0.065	3.03	0.88	0.50	1.80	6.76	1.21
80	Excimer	0.921	265	0.079	3.13	1.01	0.50	1.70	7.19	1.22

Supplementary Notes

Synthesis of TpAT-tFFO

TpAT-tFFO was prepared according to reference[1].

Synthesis of TpATtBu-tFFO

The synthesis scheme of TpATtBu-tFFO is summarized in Supplementary Figs. 13 and 14. The starting material S1 was prepared according to reference[1].

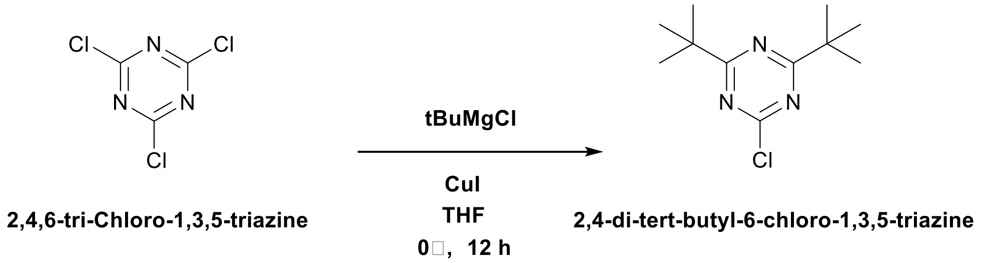

Supplementary Fig. 13. Synthesis of 2,4-di-tert-butyl-6-chloro-1,3,5-triazine.

In a 100-mL two neck round bottom flask, 2,4,6-tri-chloro-1,3,5-triazine (2.08 g, 11.3 mmol) and copper(I) iodide (70.5 mg, 0.37 mmol) were dissolved in 12 mL of dehydrated THF under an Ar atomosphere and the solution was cooled to −10 °C. After 2 h stirring, 13.6 mL (27.1 mmol) of tert-butylmagnesium chloride solution 2.0 M in THF was slowly dropwised to the stirred solution. After stirring at 0 °C for 2 h and at r.t. for 12 h, the reaction mixture was quenched with 50 ml of 2.4 M HCl aqueous solution. The resulting mixture layer was extracted with 50 mL of ethyl acetate three times. The crude mixture was concentrated under reduced pressure and then purified by silica gel column chromatography using hexane/dichloromethane = 9/1 as eluent. 2.32 g (10.2 mmol) of 2,4-di-tert-butyl-6-chloro-1,3,5-triazine (Supplementary Figure 13) was obtained in 90% yield.

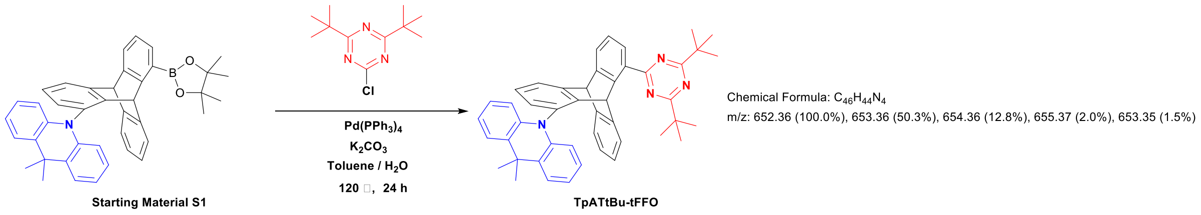

Supplementary Fig. 14. Synthesis of TpATtBu-tFFO.

A 45-mL portion of deoxidized toluene and 4.5 mL of 2 M aqueous potassium carbonate (12.0 mmol) were added to a 100-mL round bottom Schlenk flask containing Starting Material S1 (0.42 g, 0.75 mmol), 2,4-di-tert-butyl-6-chloro-1,3,5-triazine (0.29 g, 1.27 mmol), and

$Pd(PPh_3)_4$ (84 mg, 0.075 mmol). After three freeze-pump-thaw cycles under an Ar atmosphere, the mixture was stirred at 120 °C for 24 h. After the reaction mixture was cooled to ambient temperature, 40 mL of distilled water was added, and the organic layer was extracted with 50 mL of ethyl acetate three times. The combined organics were dried over sodium sulfate, concentrated under reduced pressure and then purified by column chromatography using hexane/dichloromethane = 4/1. 0.366 g (0.56 mmol) of TpATtBu-tFFO was obtained in 74% yield.

^1H NMR (400 MHz, $CDCl_3$) δ: 8.11 (dd, J = 8.0, 1.1 Hz, 1H), 7.62-7.60 (m, 1H), 7.53 (d, J = 7.2 Hz, 1H), 7.49 (d, J = 7.0 Hz, 1H), 7.43 (dd, J = 7.8, 1.4 Hz, 1H), 7.32 (d, J = 6.4 Hz, 1H), 7.23-7.15 (m, 3H), 7.13 (s, 1H), 7.10-7.06 (m, 1H), 7.03 (td, J = 7.3, 1.3 Hz, 1H), 6.98-6.94 (m, 1H), 6.86-6.81 (m, 2H), 6.55-6.51 (m, 1H), 6.38-6.34 (m, 1H), 5.84 (dd, J = 8.1, 1.0 Hz, 1H), 5.72 (dd, J = 8.1, 1.0 Hz, 1H), 5.66 (s, 1H), 1.89 (s, 3H), 1.26 (s, 3H), 1.17 (s, 18H). ^{13}C NMR (101 MHz, $CDCl_3$) δ: 184.1, 171.3, 148.1, 147.2, 146.4, 145.8, 144.6, 143.8, 140.9, 140.5, 136.1, 133.4, 130.6, 129.9, 128.5, 128.0, 126.7, 126.5, 126.2, 126.1, 125.7, 125.2, 125.2, 125.2, 123.7, 123.5, 123.4, 123.4, 120.8, 120.3, 114.8, 113.3, 77.3, 77.0, 76.7, 54.8, 45.6, 39.2, 36.1, 33.0, 28.8, 23.6. APCI-MS (m/z): [M+H]+ calcd. for $C_{46}H_{45}N_4$, 653.3639; found, 653.3636.

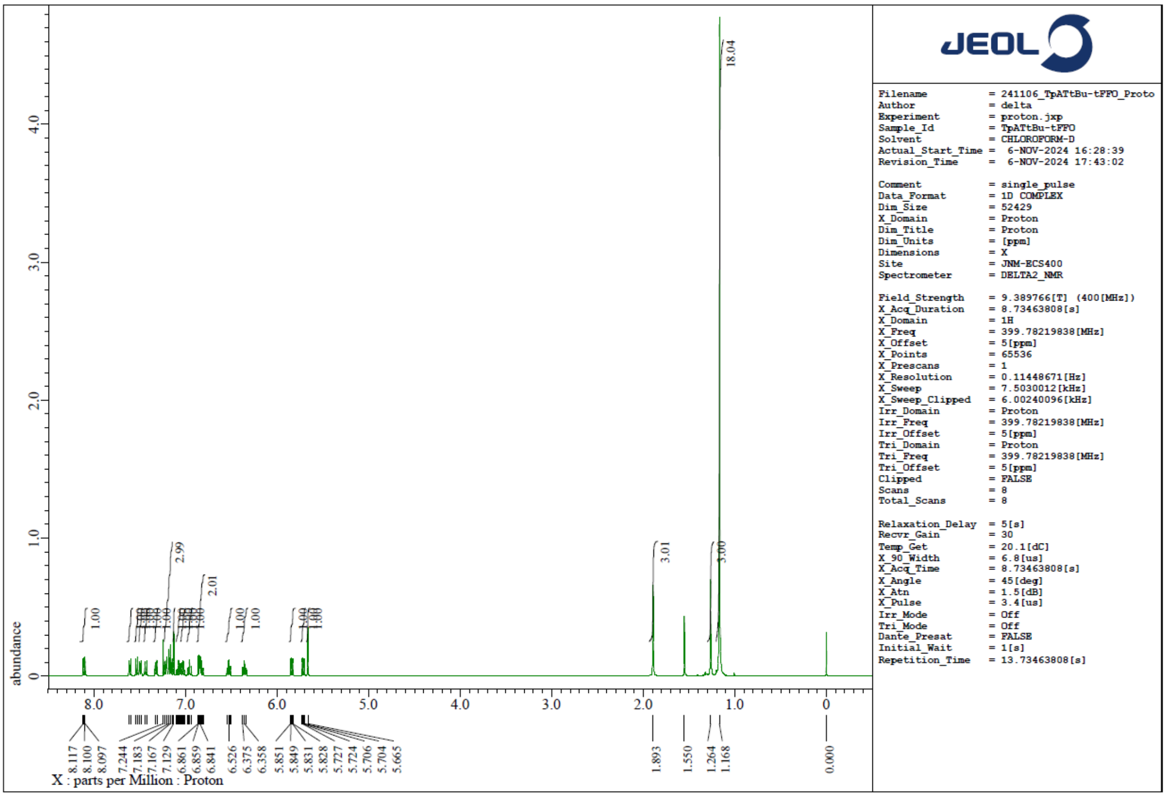

Supplementary Fig. 15. ^1H NMR spectrum of TpATtBu-tFFO.

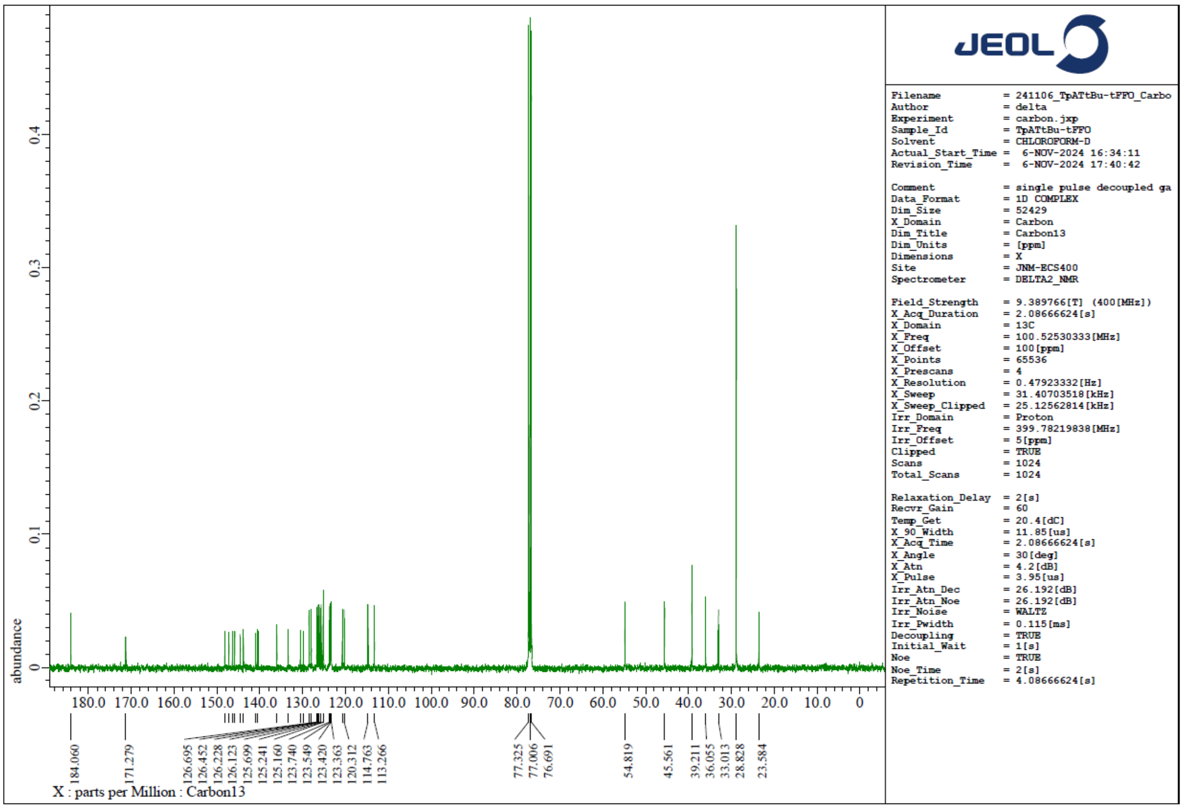

Supplementary Fig. 16. $^{13}C\{^{1}H\}$ NMR spectrum of TpATtBu-tFFO.

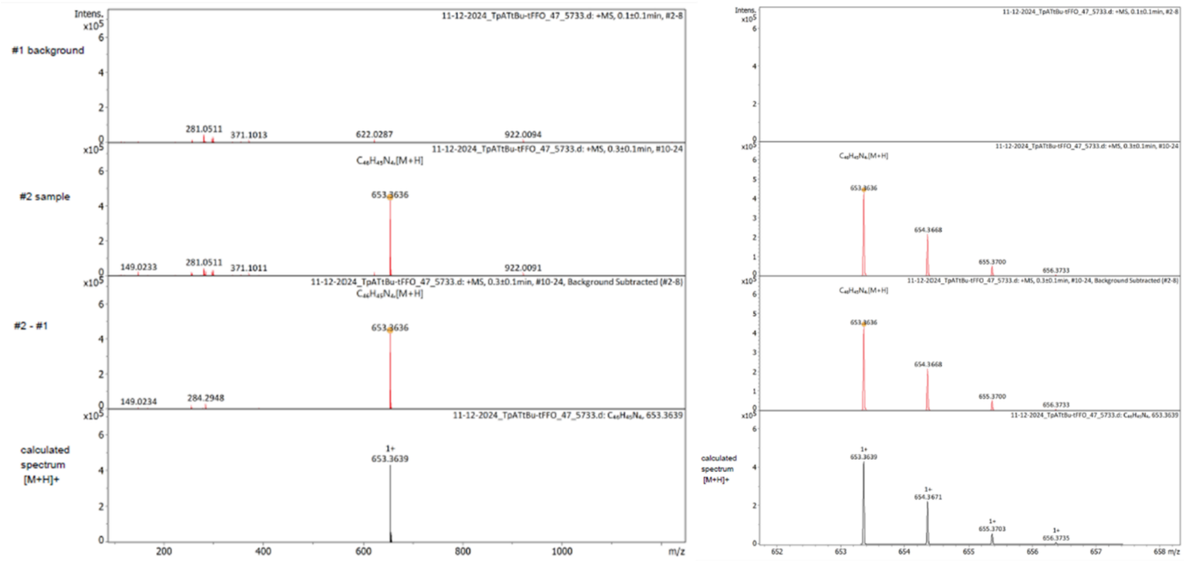

Supplementary Fig. 17. Mass spectrum of TpATtBu-tFFO.

Supplementary References

1. Wada, Y., Nakagawa, H., Matsumoto, S., Wakisaka, Y., & Kaji, H. *Nat. Photon.* **14**, 643-649 (2020).

2. Frisch, M. J. *et al*. Gaussian 16, Revision C.01, Gaussian, Inc., Wallingford CT, 2016.

3. Martínez, L., Andrade, R., Birgin, E. G., Martínez, J. M. Packmol: A package for building initial configurations for molecular dynamics simulations. *J. Comput. Chem.* **30,** 13, 2157-2164 (2009).

4. Thompson, A. P. *et al*. LAMMPS—a flexible simulation tool for particle-based materials modeling at the atomic, meso, and continuum scales. *Comput. Phys. Commun.* **271**, 108171 (2022).

5. Lu, T. A comprehensive electron wavefunction analysis toolbox for chemists, Multiwfn, *J. Chem. Phys.* **161**, 082503 (2024).
