# Supplementary material for: Electrochemically induced hyperfluorescence based on the formation of charge-transfer excimers
Source: Nat Commun. 2026 Mar 10;17:3753. doi: 10.1038/s41467-026-70291-9 (PMC13106640; doi:10.1038/s41467-026-70291-9)
Supplement: Supplementary file 2 — Description of Additional Supplementary Files [file 41467_2026_70291_MOESM2_ESM.pdf]

Description of Additional Supplementary Files

Supplementary Movie 1. Operation of calligraphic ECLD display with floating bipolar electrode configuration, featuring gold electrodes connected to each contact pad. The alligator clip on the right side is connected to a sacrificial electrode. ECL is generated on the surface of each calligraphic gold electrode by subsequently applying AC voltage to each contact pad.
